# LRRK2: an éminence grise of Wnt-mediated neurogenesis?

**DOI:** 10.3389/fncel.2013.00082

**Published:** 2013-05-31

**Authors:** Daniel C. Berwick, Kirsten Harvey

**Affiliations:** Department of Pharmacology, University College London School of Pharmacy, University College LondonLondon, UK

**Keywords:** LRRK2, Wnt signaling, neurogenesis, Parkinson’s disease, DVL, LRP6, GSK3

## Abstract

The importance of leucine-rich repeat kinase 2 (LRRK2) to mature neurons is well-established, since mutations in *PARK8*, the gene encoding LRRK2, are the most common known cause of Parkinson’s disease. Nonetheless, despite the LRRK2 knockout mouse having no overt neurodevelopmental defect, numerous lines of *in vitro* data point toward a central role for this protein in neurogenesis. Roles for LRRK2 have been described in many key processes, including neurite outgrowth and the regulation of microtubule dynamics. Moreover, LRRK2 has been implicated in cell cycle control, suggesting additional roles in neurogenesis that precede terminal differentiation. However, we contend that the suggested function of LRRK2 as a scaffolding protein at the heart of numerous Wnt signaling cascades provides the most tantalizing link to neurogenesis in the developing brain. Numerous lines of evidence show a critical requirement for multiple Wnt pathways in the development of certain brain regions, not least the dopaminergic neurons of the ventral mid-brain. In conclusion, these observations indicate a function of LRRK2 as a subtle yet critical mediator of the action of Wnt ligands on developing neurons. We suggest that LRRK2 loss- or gain-of-function are likely modifiers of developmental phenotypes seen in animal models of Wnt signaling deregulation, a hypothesis that can be tested by cross-breeding relevant genetically modified experimental strains.

## LEUCINE-RICH REPEAT KINASE 2

Leucine-rich repeat kinase 2 (LRRK2) is a protein that has been the subject of extensive research in recent years. This interest stems from the identification of LRRK2 as the product of the human *PARK8* gene, previously implicated as the cause of a familial form of Parkinson’s disease in genetic linkage studies (Paisán-Ruíz et al., 2004; Zimprich et al., 2004). Furthering interest in LRRK2, *PARK8* has subsequently been associated with cancer, leprosy, and Crohn’s disease (Hassin-Baer et al., 2009; Van Limbergen et al., 2009; Zhang et al., 2009). The importance of LRRK2 to these diseases will not be discussed further as the relevance to neuronal biology is limited. Nonetheless, the association of a single gene with four distinct medical conditions serves to highlight the complexity of LRRK2 function.

Parkinson’s disease is a currently incurable late-onset neurodegenerative disorder with increasing public health implications in an aging population (Gasser, 2010). Therefore, uncovering the molecular events causing this condition with the ultimate aim of identifying therapeutic targets for disease modifying treatment has gained in importance. The *PARK* genes mutated in patients with familial Parkinson’s disease represent an obvious starting point for this research. Although *PARK8* is just one of more than a dozen loci linked to Parkinson’s disease, certain lines of evidence indicate that LRRK2 is of special relevance. Globally *PARK8* mutations are estimated to contribute to 1–5% of Parkinson’s disease cases, which represents the greatest contribution from any known genetic or environmental cause (Kumari and Tan, 2009). In some populations, most notably North African Berbers, *PARK8* mutations are very common and account for as much as two-fifths of all Parkinson’s disease cases (Lesage et al., 2005; Jasinska-Myga et al., 2010). Importantly, patients with *PARK8* mutations exhibit symptoms that are clinically indistinguishable from the more common idiopathic form of Parkinson’s disease, while observed post-mortem brain pathologies are also largely identical (Zimprich et al., 2004). Thus, it seems likely that LRRK2 also plays a role in an as yet undetermined process that is deregulated very early in the pathogenesis of idiopathic Parkinson’s disease (Kumari and Tan, 2009; Berwick and Harvey, 2011).

**FIGURE 1:**
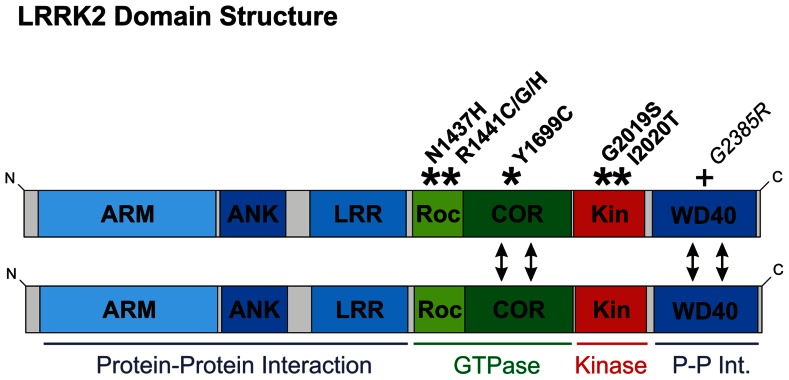
**Domain structure of LRRK2**. Domains are color-coded according to function: those implicated in protein–protein interaction are depicted in blue; domains involved in GTPase function are green; and the kinase domain red. Here, LRRK2 is depicted as a dimer, although LRRK2 also exists as monomers and in higher molecular weight complexes (Greggio et al., 2008). Dimerization is likely to be mediated by the COR and/or WD40 domains (double-headed arrows). COR domains are established as dimerization devices in ROCO proteins (Gotthardt et al., 2008), whilst ablation of the WD40 domain has been reported to disrupt LRRK2 dimerization (Jorgensen et al., 2009). The location of pathological mutations proven to segregate with Parkinson’s disease are shown with asterisks and bold font. Although only considered a risk factor, the G2385R mutation is also depicted with a plus sign, since this mutation is mentioned in the main text and is very frequent amongst Asian populations. ARM, armadillo repeat; ANK, ankyrin repeat; LRR, leucine-rich repeat; Roc, ras of complex proteins; COR, c-terminal of Roc; Kin, kinase.

From a biochemical perspective, there are two immediate observations to be made about LRRK2. First, LRRK2 is a large (2527 amino acid) protein, containing multiple protein–protein interaction domains (**Figure 1**). Unsurprisingly, a vast number of interaction partners have been reported and LRRK2 has been suggested to function primarily as scaffolding protein (Berwick and Harvey, 2011; Lewis and Manzoni, 2012). Indeed, the breadth of reported interactors is so wide that LRRK2 probably functions in a number of distinct multi-protein complexes. Second, LRRK2 contains two separate enzymatic activities: serine–threonine phosphorylation (kinase activity) and guanine triphosphate hydrolysis (GTPase activity). Understandably, this has lead to the suggestion of alternative roles for LRRK2 as a “conventional” signaling protein, either functioning as a protein kinase or in an analogous manner to small GTPases such as Ras or Rac (Berwick and Harvey, 2011).

A detailed review of LRRK2 function is beyond the scope of this article, but what is most important to stress is that the function of LRRK2 remains unclear and in many cases is controversial. For example, 8 years of research have failed to find a reproducible kinase substrate other than LRRK2 itself, while there is still no agreement on whether the GTPase activity controls kinase activity or vice versa. It seems probable therefore that these functions are interdependent. In any case, the enzymatic activities of LRRK2 are certainly of some importance in the physiological and pathological function of this protein. To date all described *PARK8* mutations clearly segregating with Parkinson’s disease cause changes in the GTPase or kinase domains, but not in any of the protein–protein interaction domains (**Figure 1**). Over-expression of LRRK2 in cultured cells and transgenic animals has been widely reported to cause cytotoxicity (Greggio et al., 2006; Smith et al., 2006; Iaccarino et al., 2007; West et al., 2007; Xiong et al., 2010; Stafa et al., 2012; Biosa et al., 2013). Whether this observation reflects an artifactual effect of over-expressing this large protein is unclear. Nonetheless, it is now generally accepted that this observed cytotoxicity is enhanced by *PARK8* mutations but ameliorated by loss of LRRK2 kinase or GTPase activity. In light of this, much work has been predicated on the idea that pathological effects of *PARK8* mutations require LRRK2 kinase activity, and in consequence a great deal of effort has gone into developing a pharmacological inhibitor of LRRK2 kinase activity (Ray and Liu, 2012). However, although the common G2019S mutation has been reproducibly shown to elicit increased LRRK2 kinase activity this mutation appears unique: all other mutations segregating with Parkinson’s disease have no reproducible effect on kinase activity (Greggio and Cookson, 2009). Furthermore, the G2385R mutation, a risk factor within Asian populations, has been reported to have decreased kinase activity (Rudenko et al., 2012). Thus LRRK2 kinase activity appears to regulate the function of this protein, but whether increased kinase activity is responsible for pathogenesis in *PARK8* patients remains to be established. In conclusion, we favor a model where LRRK2 functions primarily as a scaffold that nucleates multiple protein complexes, but where protein function is nonetheless dependent on LRRK2 kinase and GTPase activities.

**FIGURE 2:**
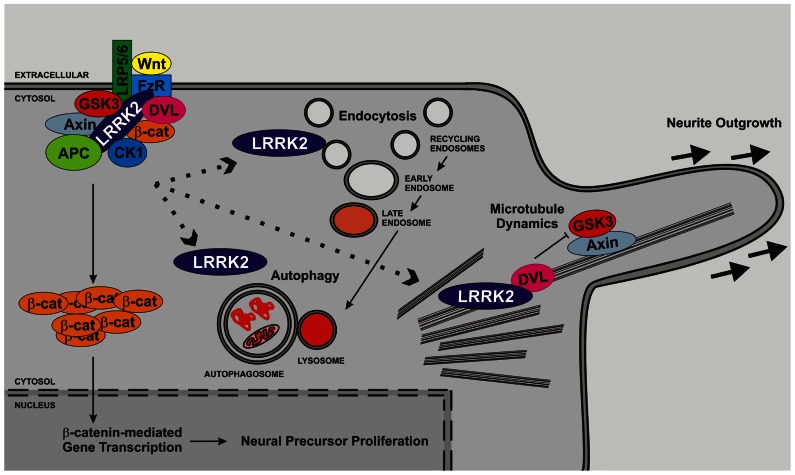
**LRRK2 regulates cell biological functions important for neurogenesis**. Several lines of evidence support roles for LRRK2 in microtubule function, endocytosis/vesicle trafficking and autophagy. LRRK2 is likely to impact upon neurite outgrowth and the latter stages of neurogenesis through direct association with membrane structures and microtubules and/or regulation of signaling pathways. In addition, LRRK2 has been implicated in proliferation and may therefore also govern early stages of neurogenesis. The interaction between microtubule function, vesicle trafficking, autophagy and proliferation and the canonical Wnt pathway is depicted but other pathways are likely to play additional roles.

Despite considerable disagreement about LRRK2 function at the biochemical level, cell biological and transgenic animal studies have allowed advances to be made. In the following section we review aspects of LRRK2 biology where there are sufficient data to paint an overall picture that is beyond dispute, even if specific details are controversial or not yet known. We value the importance of work performed in lower organisms and will mention data obtained from these systems where pertinent, however, this review will focus on mammalian data. This distinction is justified since mammals express two LRRK proteins, LRRK1 and LRRK2, which despite strong similarities in sequence and structure appear to have contrasting functions. Lower organisms, in particular *Drosophila melanogaster* and *Caenorhabditis elegans*, encode a single LRRK protein, which is thus the ortholog of both LRRK1 and LRRK2. As we outline, there is sufficient evidence in existence to make roles for LRRK2 in three processes that underlie neurogenesis beyond debate. These are roles in synaptic and endosomal vesicle trafficking, macroautophagy, and regulation of microtubule dynamics are illustrated in **Figure 2**. Furthermore, an effect of LRRK2 on neurite outgrowth – a direct measure of the latter stages of neurogenesis – is extremely well supported. Coupled with growing evidence of roles in adult neurogenesis and in proliferation we contend that these data make a strong case for a central role for LRRK2 in multiple stages of neurogenesis.

## ROLES FOR LRRK2 IN FUNCTIONS UNDERLYING NEUROGENESIS

Regulated membrane trafficking events underlie many key processes involved in neurogenesis. These include requirements for endocytosis for the proper function of neurogenic signaling pathways, such as Notch and Wnt cascades, and membrane receptors involved in axonal outgrowth (Blitzer and Nusse, 2006; Le Borgne, 2006; Winckler and Yap, 2011). Importantly, roles for LRRK2 in membrane trafficking events are supported by data extending from early reports placing LRRK2 protein on cellular membranes (Biskup et al., 2006; Hatano et al., 2007) to evidence of vacuolation in LRRK2 knockout mouse kidney cells (Tong et al., 2012). Intriguingly, the kinase activity of LRRK2 appears to be enhanced at membranes (Berger et al., 2010), while the distribution of LRRK2 between membrane and cytosolic fractions can be regulated by extracellular stimuli (Berwick and Harvey, 2012a). These observations suggest that LRRK2 plays an active role in membrane trafficking events, and is not simply present as a by-stander.

The precise membranous compartments and/or vesicles LRRK2 inhabits remains contentious since many have been suggested, however, membrane compartments involved in two cellular processes – pre-synaptic vesicle trafficking and macroautophagy – stand prominent. Evidence of a role for LRRK2 in the trafficking of pre-synaptic vesicles comes from multiple experimental techniques. These include localization of LRRK2 to vesicles in synaptic terminals by confocal and electron microscopy (Xiong et al., 2010; Piccoli et al., 2011), demonstrable electrophysiological defects following knock-down or over-expression of LRRK2 in cultured neurons (Shin et al., 2008; Piccoli et al., 2011), and biochemical interaction and co-localization of LRRK2 with the early endosomal marker Rab5b (Shin et al., 2008). The function of LRRK2 in pre-synaptic vesicular compartments remains to be determined, although a recent report that the LRRK2 homolog in *Drosophila* phosphorylates endophilin A proteins is promising (Matta et al., 2012).

A wealth of data implicate LRRK2 in macroautophagy, the process by which damaged organelles and protein aggregates are “consumed” by engulfment by membranous autophagosomes that subsequently fuse with lysosomes (Codogno et al., 2012). Macroautophagy is traditionally considered a mechanism of cellular homeostasis and regulated cell death. However, neural development is especially dependent on this process, most likely for mediating the extensive physical remodeling required (Cecconi et al., 2007; Cecconi and Levine, 2008). Studies performed using mouse models of LRRK2 dysfunction have been particularly revealing, since multiple observations show impaired macroautophagy in the kidney of *Lrrk2* null animals (Tong et al., 2010, 2012; Herzig et al., 2011). These data include increased numbers of lysosomes and related structures, and an accumulation of the macroautophagy substrates p62 protein and lipofuscin granules. Much of this kidney phenotype appears to be replicated in transgenic mice that over-express LRRK2 containing an artificial kinase-inactivating mutation (Herzig et al., 2011). In agreement with these observations, over-expression of LRRK2 impacts upon the autophagic pathway in human embryonic kidney 293 (HEK293) cells (Alegre-Abarrategui et al., 2009; Gómez-Suaga et al., 2012). Importantly, published data also support a role for LRRK2 in macroautophagic processes in the central nervous system. Most notably, brains from aged transgenic mice that over-express human LRRK2 variants containing either the R1441C or G2019S *PARK8* mutations have an accumulation of autophagic vacuoles (Ramonet et al., 2011). This observation appears to be corroborated *in vitro*, where over-expression of G2019S has similar effects in cultured primary neurons or differentiated SH-SY5Y cells (MacLeod et al., 2006; Plowey et al., 2008).

Studies in *Drosophila* suggest another connection between LRRK2 and macroautophagy: via interaction with the small GTPase Rab7 (Dodson et al., 2012). Rab7 is well-established as a key regulator of the fusion step between macroautophagic organelles and lysosomes (Codogno et al., 2012). If this observation can be replicated in mammalian systems it would be a fascinating result, since Rab7 is also involved in the latter stages of endocytosis, mediating late endosomal maturation and fusion with the lysosome (Wang et al., 2011). As mentioned above, LRRK2 has also been reported to interact with Rab5b (Shin et al., 2008). Since Rab5 proteins link pre-synaptic vesicle trafficking with early stages of endocytosis and Rab7 links autophagy with late-stage endocytosis, these observations together connect LRRK2 with the entire endocytic pathway.

In neurons endocytic vesicles can be trafficked over huge distances, particularly in axons. The importance of microtubules for this process is well described (Perlson et al., 2010). It is therefore interesting that an association between LRRK2 and microtubules has been reported by a large number of groups (Biskup et al., 2006; Gloeckner et al., 2006; Gandhi et al., 2008; Gillardon, 2009a,b; Sancho et al., 2009; Dzamko et al., 2010; Kawakami et al., 2012; Kett et al., 2012; Sheng et al., 2012). This physical association appears highly relevant to neurogenesis as LRRK2 has been reported to co-localize with microtubules within growth cones (Sancho et al., 2009).

The nature of the LRRK2-microtubule interaction is still unresolved. For example, there are conflicting reports about whether inhibition of LRRK2 kinase activity promotes (Dzamko et al., 2010) or weakens (Kett et al., 2012) the association. However, a role for LRRK2 in microtubule dynamics seems beyond dispute. Certain clues suggest LRRK2 may affect the stability of microtubules. LRRK2 has been reported to enhance the polymerization of bovine tubulin in the presence of microtubule-associated proteins (MAPs) *in vitro* (Gillardon, 2009b). In addition, LRRK2 has been linked to canonical Wnt signaling (Sancho et al., 2009; Lin et al., 2010; Berwick and Harvey, 2012a), which is well described as a modulator of the microtubule cytoskeleton in neurons (Salinas, 2007). Perhaps most strikingly though, numerous lines of *in vivo* data implicate LRRK2 in modulating the function of the MAP tau, best known for its role in Alzheimer’s disease (Taymans and Cookson, 2010). Post-mortem analysis of Parkinson’s disease brains carrying Y1699C, G2019S, or I2020T *PARK8* mutations have been reported to display “tau pathology” in a number of cases (Zimprich et al., 2004; Khan et al., 2005; Rajput et al., 2006; Ujiie et al., 2012). Tau hyperphosphorylation has also been reported in brains from transgenic mice over-expressing LRRK2 with the G2019S or R1441G mutations (Li et al., 2009; Melrose et al., 2010), while *Lrrk2* knockout has been reported to decrease tau phosphorylation (Gillardon, 2009b) although others have been unable to replicate this observation (Hinkle et al., 2012). G2019S LRRK2 has also been reported to promote tau phosphorylation in *Drosophila* (Lin et al., 2010). Mechanistically, the details linking LRRK2 to tau phosphorylation are lacking but one might predict the interaction between LRRK2 and microtubules would bring LRRK2 and tau into proximity. Indeed, a recent report suggests this may be the case (Kawakami et al., 2012). Whether LRRK2 phosphorylates tau directly remains unclear with one study supporting direct phosphorylation (Kawakami et al., 2012), and another suggesting that phosphorylation is performed by glycogen synthase kinase 3 (GSK3; Lin et al., 2010), a reported LRRK2 interactor (Lin et al., 2010; Berwick and Harvey, 2012a). These reports are not necessarily in conflict, since the experimental systems used are different and tau contains over 80 reported phosphorylation sites (http://cnr.iop.kcl.ac.uk/hangerlab/tautable). However, the possibility that phosphorylation is via GSK3 is intriguing, since this kinase has been implicated in the control of multiple MAPs besides tau, such as *adenomatous polyposis coli* (APC; Zhou et al., 2004) and collapsin response mediator protein 2 (Cole et al., 2004). Therefore, it is plausible that the control of microtubule dynamics by LRRK2 involves the modulation of a multitude of MAPs and takes place at a variety of microtubule sites, not just those regulated by tau in axons.

Parallel to a role for LRRK2 on microtubules, LRRK2 has also been connected to the actin cytoskeleton via the regulation of ERM (ezrin–radixin–moesin) protein phosphorylation. ERM proteins are three homologous proteins involved in anchoring actin filaments to the plasma membrane (Mangeat et al., 1999). Phosphorylation of ERM proteins is believed to induce a conformational change resulting in an open “active” shape (Mangeat et al., 1999). Two groups have found a positive correlation between LRRK2 levels and ERM protein phosphorylation (Jaleel et al., 2007; Parisiadou et al., 2009), that is most likely mediated by an indirect regulation (Nichols et al., 2009). Importantly, ERM proteins are essential for growth cone morphology and motility (Paglini et al., 1998), thus indicating that LRRK2 also impacts upon neurogenesis through ERM proteins. The connection between LRRK2 and the actin cytoskeleton is strengthened by a mass spectrometry study which found endogenous LRRK2 in HEK293 cells to associate with actin and a number of proteins known to modulate the actin cytoskeleton (Meixner et al., 2011). In light of these observations, it is interesting to speculate about a role for LRRK2 in coordinating neuronal microtubule as well as actin networks.

Thus, there are several lines of evidence for the importance of LRRK2 in a number of processes that underlie neurogenesis, but what evidence *directly* supports a requirement for LRRK2 in neurogenesis? Importantly, a comprehensive study in mouse embryos found a spatio-temporal LRRK2 mRNA expression pattern that is highly consistent with a key role for LRRK2 in neurogenesis (Zechel et al., 2010). Using *in situ* hybridization, LRRK2 mRNA expression was detected as early as day E10.5 in the developing central nervous system, with transcript detectable throughout the cortex by day E12.5. Crucially, the authors describe embryonic expression of Lrrk2 as being most prominent in brain regions with “high proliferative and migratory activity, as well as sites of differentiation and cell death” (Zechel et al., 2010). These include the ventricular and subventricular zones of the telencephalon, in agreement with a previous report investigating older mice (Melrose et al., 2007). Lrrk2 was also found to be expressed in neural stem cells isolated from the dentate gyrus or striatal subventricular zone of E18.5 or adult mice. Thus LRRK2 expression patterns are consistent with a role in neurogenesis throughout life.

It is thus unsurprising that a growing body of experimental data show defects in neurogenesis caused by altered LRRK2 function. Experiments in cell and animal models utilized LRRK2 knock-down or knockout, over-expression of wild-type LRRK2, familial LRRK2 mutants and artificial kinase or GTPase dead mutants to measure predominantly neurite outgrowth and reported changes in length, number and branching of neurites *in vitro* and in brain slices. Most reports agree that the over-expression of familial LRRK2 mutants elicit decreased neurite length (MacLeod et al., 2006; Plowey et al., 2008; Parisiadou et al., 2009; Dächsel et al., 2010; Heo et al., 2010; Lin et al., 2010; Chan et al., 2011; Ramonet et al., 2011; Winner et al., 2011; Maekawa et al., 2012; Sheng et al., 2012; Stafa et al., 2012; Biosa et al., 2013; Cherra et al., 2013; Cho et al., 2013). Over-expression of wild-type LRRK2 was generally found to have no effect, or to cause mild neurite shortening, either reaching statistical significance or remaining as a trend. In contrast, loss of LRRK2 appears to have the opposite effect, allowing longer and more branched neurites to develop (MacLeod et al., 2006; Parisiadou et al., 2009; Dächsel et al., 2010; Heo et al., 2010; Stafa et al., 2012; Paus et al., 2013), although not all studies are in agreement (Gillardon, 2009b; Meixner et al., 2011). In general, these studies provide overwhelming evidence that the level of LRRK2 expression impacts upon neuritogenesis as predicted from the LRRK2 expression pattern throughout brain development.

Finally, we also note two publications investigating a role for LRRK2 in adult neurogenesis (Winner et al., 2011; Paus et al., 2013). These studies are of special relevance to Parkinson’s disease, where impaired adult neurogenesis has been implicated in the development of non-motor symptoms (Marxreiter et al., 2013). A study in mice over-expressing the G2019S LRRK2 mutant reported decreased proliferation in the dentate gyrus and subventricular zones associated with decreased dendritic length and branching, and decreased cell survival (Winner et al., 2011). Another study by Paus et al. (2013) looked at adult neurogenesis in the dentate gyrus of *Lrrk2* knockout mice. As might be predicted, increased dendritic length and neurite arborization was observed. No defects in cell proliferation or survival were found however, although loss of Lrrk2 led to a greater number of doublecortin positive cells (Paus et al., 2013).

Thus a wealth of data implicates LRRK2 as a central player in the latter stages of neurogenesis – in particular neurite outgrowth and synaptogenesis – possibly via a combination of modulating vesicle trafficking and cytoskeleton dynamics. Nonetheless, emerging evidence also links LRRK2 to earlier stages of neuronal development, prior to cell cycle exit (Winner et al., 2011), supported by evidence of effects of LRRK2 on proliferation (Milosevic et al., 2009; Liu et al., 2012) and carcinogenesis (Hassin-Baer et al., 2009). Thus, LRRK2 might play a role from early mitotic neuronal precursors to terminal differentiation. As outlined above, we would suggest that LRRK2 is likely to function as a scaffolding protein in a number of distinct complexes, some of these important in neurogenesis. Nonetheless, two crucial questions remain: what is upstream of these complexes, and how are they regulated?

## A ROLE FOR LRRK2 IN Wnt SIGNALING

Wnt (wingless/Int) signaling pathways constitute a family of highly conserved signal transduction cascades that have long been established as master regulators of animal development (Freese et al., 2010). The relevance of these pathways to neurogenesis is beyond doubt and will not be reviewed in detail in this article. Nonetheless the reader should be aware that a growing body of data also implicates Wnt signaling in the function of mature, post-mitotic neurons (Inestrosa and Arenas, 2010). Moreover, deregulated Wnt signaling pathways are suggested pathomechanisms for a number of neurological conditions, including Alzheimer’s disease, Parkinson’s disease, autism, and schizophrenia (De Ferrari and Moon, 2006; Inestrosa and Toledo, 2008; Berwick and Harvey, 2012b). Thus Wnt cascades can be considered essential for the central nervous system at all stages of life.

**FIGURE 3:**
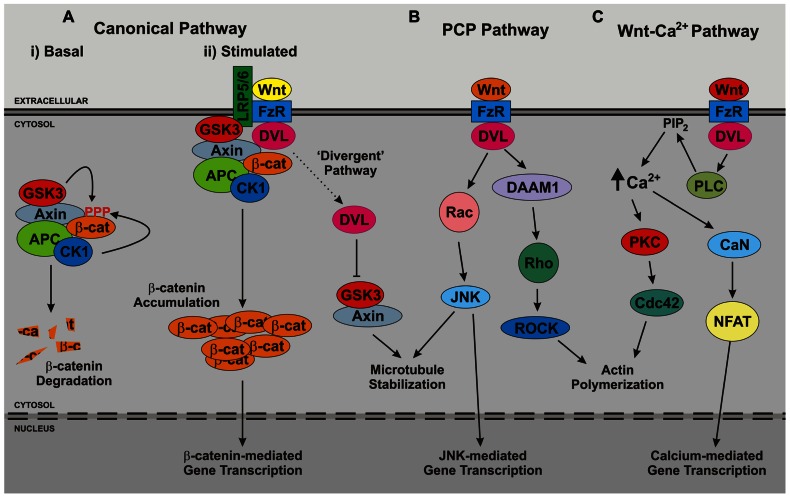
**Overview of Wnt signaling pathways**. The three major branches of Wnt signaling – **(A)** the canonical, **(B)** planar cell polarity (PCP), **(C)** and Wnt-Ca^2^^+^ – pathways are illustrated. Note that in growing neurites a further branch has been reported, the so-called divergent canonical pathway, which impacts upon microtubule stability. APC, adenomatous polyposis coli; CaN, calcineurin; CK1, casein kinase 1; DAAM, dishevelled-associated activator of morphogenesis; DVL, dishevelled; FzR, frizzled receptor; GSK3, glycogen synthase kinase 3; JNK, c-Jun n-terminal kinase; LRP5/6, low-density lipoprotein receptor-related protein 5/6; NFAT, nuclear factor of activated T cells; PKC, protein kinase C; PLC, phospholipase C; ROCK, Rho-associated protein kinase.

Wnt ligands themselves are secreted glycoproteins that bind to the extracellular domains of frizzled receptors on the plasma membrane of target cells. Signaling specificity is achieved in part through the large repertoire of Wnt ligands and frizzled receptors expressed in higher organisms, but also through the involvement of co-receptors. In the case of the canonical Wnt pathway, these receptors are low-density lipoprotein receptor-like proteins 5 and 6 (LRP5/6), which have also been reported to bind Wnt ligands at the cell surface. Upon binding of Wnt ligands to frizzled receptors and associated co-receptors the signal is relayed across the membrane, resulting in the activation of one or more intracellular cascades. Wnt signaling pathways relevant to neurogenesis and/or the function of mature neurons are depicted in **Figure 3**.

The best-described Wnt signaling cascade is the canonical Wnt pathway. This signaling mechanism ultimately results in the activation and nuclear recruitment of β-catenin protein, leading to the modulation of downstream target genes. In consequence, this pathway is sometimes referred to as the Wnt-β-catenin pathway. Canonical Wnt signaling is an unusual signaling mechanism as several events take place in the absence of a stimulus. In particular, β-catenin is sequestered into an inhibitory cytosolic complex known as the β-catenin destruction complex. Here, β-catenin is phosphorylated by GSK3 (the same protein implicated in tau phosphorylation). β-catenin phosphorylation results in the targeting of β-catenin for degradation by the proteasome. Therefore, in the absence of canonical Wnt pathway activators, β-catenin is continually degraded, and consequently unable to accumulate in the nucleus to regulate gene expression. Binding of Wnt ligand to frizzled receptors and LRP5/6 results in the recruitment of cytosolic dishevelled (DVL) proteins (key intermediates of most Wnt signaling branches) to the plasma membrane. Via interaction with key components of the β-catenin destruction complex such as Axin, DVL proteins cause the subsequent relocalization of the β-catenin destruction complex to the same juxtamembrane site. This elicits the penultimate stage of canonical Wnt signaling, the inhibition of β-catenin phosphorylation, which allows β-catenin to become proteasome resistant and thus accumulate throughout the cell. However, the complexity of this mechanism is emerging. A requirement for the internalization of the cell membrane-associated protein complex containing Wnt ligand, frizzled receptor, LRP5/6, DVL proteins, and the β-catenin destruction complex into the endosomal system is now widely accepted. This internalized signaling complex passes through the endosomal system where it continues to signal from the cytosolic face of intracellular membranes (the so-called “signalosome” hypothesis). Finally, the signalosomes are sequestered from the cytosol into multi-vesicular bodies. Traditionally, by analogy to growth factor signaling pathways, this has been considered the termination step in Wnt signaling. However, recent data suggest that the sequestration of signalosomes into multi-vesicular bodies constitutes a final “signal activation” mechanism, since this leads to the removal of the canonical Wnt signaling pool of GSK3 from the cytosol (Dobrowolski and De Robertis, 2012).

Data from our laboratory has strongly implicated LRRK2 in multiple aspects of the canonical Wnt pathway. This work arose from a yeast two-hybrid screen identifying interactors of the Roc and/or COR domains of LRRK2 (Sancho et al., 2009). Since Roc and COR domains are expressed together throughout nature (Marín et al., 2008), it is reasonable to consider both domains part of a single functional unit conferring GTPase activity that was termed the RocCOR tandem domain. Using this LRRK2 RocCOR tandem domain as bait, the yeast two-hybrid screen returned cDNAs encoding the human DVL proteins DVL2 and DVL3 as potential interactors. Subsequent assays confirmed a direct interaction between the LRRK2 RocCOR domain and all three human DVL proteins, with the interaction site mapped to the DVL–Egl10–pleckstrin (DEP) domain of DVL1–3 (Sancho et al., 2009). Co-immunoprecipitation experiments confirmed that LRRK2 associates with DVL proteins in mammalian cells, while confocal microscopy revealed a striking recruitment of LRRK2 into polymeric DVL structures that are induced by over-expression of these proteins (Sancho et al., 2009).

Since DVL proteins are essential intermediates of all major branches of Wnt signaling (**Figure 3**), our work opened the possibility that LRRK2 may function in multiple Wnt cascades. However, follow-up studies focused on the canonical Wnt pathway, which can be assayed easily using TOPflash assays (Veeman et al., 2003). TOPflash assays are luciferase-based reporter assays that quantitatively determine the level of β-catenin-mediated transcriptional activity in cells. Importantly, TOPflash assays revealed the LRRK2–DVL protein interaction to be functional as well as physical, since co-transfection of LRRK2 protein with any of the three human DVL proteins resulted in an enhancement of DVL-driven canonical Wnt activity (Berwick and Harvey, 2012a). This effect required the kinase and GTPase activities of LRRK2 and was increased further by targeting LRRK2 (and presumably, therefore, the LRRK2–DVL interaction) to membranes. Interestingly, Wnt3a treatment was found to increase the amount of endogenous LRRK2 present in membrane fractions of HEK293 cells (Berwick and Harvey, 2012a). Since the activation of canonical Wnt signaling takes place at intracellular membranes – consistent with the signalosome hypothesis – this lead us to investigate whether LRRK2 might physically interact with Wnt signaling receptors. Using a combination of confocal microscopy and co-immunoprecipitation LRRK2 was discovered to associate with LRP6, but not frizzled-1, frizzled-4, or frizzled-5. Yeast two-hybrid assays confirmed that the interaction was direct and, similar to interaction with DVL proteins, involved the LRRK2 RocCOR tandem domain (Berwick and Harvey, 2012a). These data are consistent with a role for LRRK2 in the activation of canonical Wnt signaling bringing DVL proteins to cellular membranes.

However, somewhat counter-intuitively, knock-down of LRRK2 was also found to potentiate DVL-driven TOPflash activity (Berwick and Harvey, 2012a). A similar effect was observed on basal and Wnt3a-driven β-catenin activity. In this regard knock-down of LRRK2 mimicked knock-down of AXIN1, an established component of the β-catenin destruction complex. Loss of AXIN1 is well-known to disrupt the β-catenin destruction complex thereby compromising β-catenin degradation and leading to an increase in basal canonical Wnt activity. We therefore wondered whether loss of LRRK2 might compromise an inhibitory role for LRRK2 in the β-catenin destruction complex. Consistent with this, co-immunoprecipitation of endogenous protein from mouse brain revealed Lrrk2 to exist in complex with multiple components of the β-catenin destruction complex, including GSK3 and β-catenin. Taken together, these results suggest a role for LRRK2 as a scaffold in canonical Wnt signaling. In the basal state, LRRK2 functions as part of the cytosolic β-catenin destruction complex and loss of LRRK2 compromises this role, leading to disruption of the complex and pathway activation. Following stimulation of cells with Wnt ligand, LRRK2 is recruited to cellular membranes. Here, via interaction with DVL proteins, the β-catenin destruction complex and LRP6, LRRK2 assists in the formation of Wnt signalosomes, enhancing the Wnt signal activity.

Our data are supported by work from other laboratories. Most notably, supporting the idea that LRRK2 associates with the β-catenin destruction complex, an interaction between LRRK2 and GSK3 has been reported in *Drosophila* (Lin et al., 2010). While this report does not investigate which cellular GSK3 pool associates with LRRK2 we note that the interaction modulated tau phosphorylation. Since Wnt signaling is well-known to regulate the phosphorylation of tau by GSK3 (Hooper et al., 2008), this would suggest that the GSK3 pool found to bind LRRK2 by Lin and colleagues could indeed represent the same Wnt-responsive fraction identified in our study (Lin et al., 2010; Berwick and Harvey, 2012a). Perhaps most interesting in this report, the LRRK2–GSK3 interaction was shown to be enhanced by the G2019S *PARK8* mutation (Lin et al., 2010). Curiously, the strength of the LRRK2 interaction with both DVL proteins and LRP6 was also affected by *PARK8* mutations (Sancho et al., 2009; Berwick and Harvey, 2012a). Unsurprisingly therefore, all investigated *PARK8* mutations decreased the capacity of LRRK2 to enhance the β-catenin activation elicited by DVL proteins (Berwick and Harvey, 2012a). This observation has obvious implications for the pathogenesis of Parkinson’s disease, where perturbed Wnt signaling has already been suggested as a candidate pathomechanism (Berwick and Harvey, 2012b). However, as we outline below, decreased canonical Wnt signaling associated with familial *PARK8* mutations suggests that transgenic LRRK2 animal models of Parkinson’s disease might present with discrete developmental phenotypes associated with Wnt dysfunction.

In addition to studies linking LRRK2 to Wnt signaling by protein–protein interaction strong circumstantial evidence from transcriptomics studies support this notion. In particular, an investigation into the effect of LRRK2 knock-down in human SH-SY5Y cells found mRNA species encoding a number of Wnt signaling proteins to be altered (Häbig et al., 2008). As it is well described that many Wnt signaling components are regulated at the transcriptional level by pathway activation, knock-down of LRRK2 would be expected to alter expression of other Wnt signaling proteins. Data from *C. elegans* also support this observation, with mRNA transcripts encoding Wnt signaling proteins being described as “coregulated with LRRK2” (Ferree et al., 2012).

Further support, albeit indirect, of a role for LRRK2 in Wnt signaling comes from studies investigating altered gene expression in animal models of Parkinson’s disease. These investigations used a variety of different neurotoxins to elicit dopaminergic cell death resulting in parkinsonian-like motor phenotypes. L’Episcopo et al. (2011) reported increased *Wnt1* gene expression as well as deregulated *Fzd1* and β-catenin expression in the ventral mid-brain of 1-methyl-4-phenyl-1,2,3,6-tetrahydropyridine (MPTP)-treated mice. These experiments support the idea that canonical Wnt signal activation via increased *Wnt1* expression in astrocytes is neuroprotective (L’Episcopo et al., 2011). Another study used 6-hydroxydopamine (6-OHDA) to induce dopaminergic cell death in rats resulting in increased expression of the Wnt signal inhibitor Dickkopf-1 (Dkk1; Dun et al., 2012). Both models are in agreement with a neuroprotective role for Wnt signaling, since treatment with Dkk1 exacerbated toxic effects, whilst GSK3 inhibition was found to be protective (L’Episcopo et al., 2011; Dun et al., 2012). An unbiased genome-wide RNAseq approach in mice treated with a variety of pesticides showed altered expression of mRNAs encoding Wnt signaling components in ventral mid-brain and striatum (Gollamudi et al., 2012). Exposure to pesticides is a well-known environment risk factor for Parkinson’s disease, further suggesting that dysregulated Wnt signaling might be a common mechanism underlying dopaminergic cell death in Parkinson’s disease. Expression studies in human Parkinson’s disease brains have not been as conclusive, although it is important to note that altered expression of Wnt pathway genes has been reported in women but not in men (Cantuti-Castelvetri et al., 2007). These studies need to take into account that the brains analyzed are usually from individuals with symptomatic Parkinson’s disease reflecting the loss of the majority of dopaminergic neurons in the substantia nigra. Therefore, gene expression changes are likely no longer reflective of the initial underlying etiology. However, in addition to work on LRRK2 outlined above, other clues from genetic causes of Parkinson’s disease are consistent with altered Wnt signaling. Most notably Parkin, the product of the *PARK2* gene, has been reported to inhibit canonical Wnt signaling (Rawal et al., 2009), whilst the transcription factor Nurr1, which has been strongly linked to Parkinson’s disease, is regulated by β-catenin (Jankovic et al., 2005; Kitagawa et al., 2007). Finally, it is not just Parkinson’s disease-related genes that have been associated with Wnt signaling; Wnt signaling genes themselves have been linked to risk of Parkinson’s disease. In particular, GSK3β has been suggested to modify disease risk in two studies (Kwok et al., 2005; Kalinderi et al., 2011) although a third failed to find any affect (Wider et al., 2011).

In summary, therefore, LRRK2 binds three central Wnt signaling components (Sancho et al., 2009; Lin et al., 2010; Berwick and Harvey, 2012a), while loss of LRRK2 and pathogenic *PARK8* mutations impact upon the activity of the canonical Wnt pathway (Berwick and Harvey, 2012a). In addition, connections between LRRK2 and Wnt cascades are strengthened by a number of studies supporting a role for dysregulated Wnt signaling in the early stage of Parkinson’s disease. As outlined above, there is overwhelming evidence for a central function for LRRK2 in neurogenesis. Combining these ideas, we postulate a specific role for LRRK2 in Wnt-mediated neurogenesis. In the final section of this article, we will elaborate on this and suggest experimental approaches to test our hypothesis.

## LRRK2 AS A MAJOR PLAYER IN Wnt-MEDIATED NEURONAL DIFFERENTIATION?

It is beyond dispute that Wnt ligands represent potent morphogens required for numerous aspects of neurogenesis, in particular the development of dopaminergic neurons of the ventral mid-brain (Brault et al., 2001; Castelo-Branco et al., 2010, 2004, 2003, 2010; Parish et al., 2008; Carpenter et al., 2010). Degeneration of these neurons underlies the typical motor symptoms associated with Parkinson’s disease (Berwick and Harvey, 2012b). In this context, deregulated Wnt signaling caused by *PARK8* mutations might cause subtle defects in establishing neuronal circuitries, leaving these dopaminergic neurons more vulnerable to additional insults important for the pathogenesis of Parkinson’s disease. In the remainder of this review, we describe roles for Wnt signaling pathways in modulating the same neurogenic events that were reported to be influenced by LRRK2. Combined with evidence of a function for LRRK2 as a Wnt signaling scaffold, this further supports the idea that LRRK2 is a central player in Wnt-mediated neurogenesis.

Evidence that Wnt ligands are major regulators of synaptic vesicle trafficking and synaptogenesis is accumulating (Farías et al., 2010; Inestrosa and Arenas, 2010). Published data support pre-synaptic and post-synaptic effects of multiple branches of Wnt signaling. Mammalian pre-synaptic development appears particularly dependent on Wnt-7a, an agonist of the canonical Wnt pathway (Farías et al., 2010). This Wnt ligand appears to be required for normal expression of the pre-synaptic vesicle protein, synapsin 1, in the developing mouse brain (Hall et al., 2000), with similar effects seen in mature neurons (Farías et al., 2007). Treatment of cultured neurons with Dkk1, an LRP5/6 antagonist, has confirmed this pre-synaptic effect of Wnt7a is through the canonical pathway (Davis et al., 2008). Curiously though, data from a number of laboratories suggest this effect is independent of transcription (Farías et al., 2010). This observation has led to the cascade by which Wnt7a modulates pre-synaptic and axonal (see below) function being described as a “divergent” canonical cascade (Ciani et al., 2004). Interestingly, LRRK2 is not just a key Wnt signaling protein interacting with LRP6 but was also found to interact with synapsin 1 and play a role in synaptic vesicle trafficking (Piccoli et al., 2011). The above evidence supports the idea of a Wnt7a-induced LRRK2-mediated canonical Wnt pathway with a direct transcriptionally independent effect on synapse formation and maintenance.

Both LRRK2 and Parkinson’s disease pathogenesis have been linked to macroautophagy. Importantly, there is also evidence consistent with the idea that macroautophagy is modulated by Wnt ligands. Strikingly, knock-down of β-catenin alone appears sufficient to induce macroautophagy in carcinoma cells (Chang et al., 2013). Correspondingly, acute treatment of hippocampal neurons with the β-catenin agonist 2-amino-4-[3,4-(methylenedioxy)benzyl-amino]-6-(3-methoxyphenyl)pyrimidine (Liu et al., 2005) was found to reduce oxygen–glucose deprivation-induced macroautophagy (Wang et al., 2012). Taken together, these observations suggest β-catenin to be a negative regulator of macroautophagy. In addition, GSK3 activity almost certainly impacts upon macroautophagy. GSK3 has recently been reported to phosphorylate TIP60, a histone acetyl transferase required for induction of macroautophagy. Mutation of the reported phosphorylation site to an alanine residue is sufficient to prevent growth factor deprivation-induced macroautophagy (Lin et al., 2012). In agreement with this, intranasal treatment with a GSK3 inhibitor peptide was reported to result in decreased autophagy and increased lysosomal acidification in brains from an Alzheimer’s disease transgenic mouse model (Avrahami et al., 2013), with similar results obtained *in vitro*. These data predict a model where canonical Wnt pathway activation – resulting in GSK3 inhibition and β-catenin accumulation – would lead to decreased macroautophagy. However, the events do not appear straightforward, since GSK3 inhibition in a neuroblastoma cell line has been reported to induce increased lysosomal biogenesis, leading to increased macroautophagic flux (Parr et al., 2012). There are numerous reasons for this potential discrepancy, for example, cell lines and treatments used, however it is perhaps more relevant to observe that these studies are at very early stages. More pertinently still, none look at the regulation of macroautophagy during neural differentiation, where one would expect the requirements placed on the autophagic machinery of developing neurons to be more subtle than under conditions of stress. In conclusion, even though evidence supports the importance for LRRK2 and canonical Wnt signaling in macroautophagy, the specific signal transduction cascade, especially during neuronal differentiation, requires further investigation.

Wnt signaling is well-known to influence the dynamic instability of the microtubule cytoskeleton (Salinas, 2007). Multiple proteins involved in both the canonical and non-canonical Wnt pathway have been reported to affect microtubule stability, whilst GSK3 phosphorylates a variety of MAPs (Cole et al., 2004; Zhou et al., 2004; Salinas, 2007; Kim et al., 2011). Modulation of microtubule structures by Wnt pathways appear common to multiple cell types, for example Wnt ligands appear to be key regulators of mitotic spindles (Walston et al., 2004). In light of the above-described pre-synaptic function of LRRK2 and Wnt7a, it is important to emphasize that a large number of studies showing Wnt-mediated regulation of microtubules have used axon outgrowth as the model system. Much of this work was initiated by the observation that Wnt7a elicits axonal spreading and branching in cultured cerebellar granule cells (Lucas and Salinas, 1997), with corroborating data soon obtained *in vivo* (Hall et al., 2000). This effect is mimicked by GSK3 inhibitors and likely involves inhibition of phosphorylation of the microtubule-associated protein MAP1B (Lucas and Salinas, 1997; Lucas et al., 1998). Additional mechanisms involved in Wnt-mediated control of axonal microtubules include the identification of the β-catenin destruction complex members APC and AXIN1 as microtubule binding proteins in axons (Ciani et al., 2004; Purro et al., 2008). The precise details are still being elucidated, but it is fair to assume that, via interaction with microtubules, APC and AXIN1 create a spatially controlled signaling mechanism, specific to growing axons and growth cones. Importantly, LRRK2 also interacts with microtubules, induces hyperphosphorylation of the axonal MAP tau (Biskup et al., 2006; Gloeckner et al., 2006; Gandhi et al., 2008; Gillardon, 2009a,b; Sancho et al., 2009; Dzamko et al., 2010; Kawakami et al., 2012; Kett et al., 2012; Sheng et al., 2012), interacts with components of the β-catenin destruction complex (Lin et al., 2010; Berwick and Harvey, 2012a) and co-localizes with DVL1 to neurites in cell culture models at early stages of differentiation (Sancho et al., 2009). This further supports the idea of a LRRK2 mediated Wnt signaling pathway important during neuronal differentiation.

Above we have established good evidence for roles of LRRK2 and Wnt signaling in the regulation of pre-synaptic vesicle trafficking and microtubule dynamics, processes crucial for axonal outgrowth and synaptogenesis. While the evidence of a role for LRRK2 in macroautophagy is overwhelming, data supporting a role for Wnt signaling in modulating this process are more circumstantial. Nonetheless, the hypothesis that LRRK2 might function specifically in Wnt-mediated neuritogenesis is plausible, especially for the latter stages of neurogenesis. But what about the earlier stages? Here, the role for Wnt signaling is beyond doubt. For example, treatment with Wnt1, which activates the canonical Wnt pathway, causes expansion of ventral mid-brain precursors (Castelo-Branco et al., 2003). Conversely, loss of *Wnt1* in mice leads to a complete failure of mid- and hind-brain precursors to expand, leading to a near absence of these brain regions (Thomas and Capecchi, 1990). Similarly, Wnt3a (another canonical pathway agonist) secreted by hippocampal astrocytes has been shown to be essential for adult neurogenesis in the dentate gyrus (Lie et al., 2005). By contrast evidence of a function for LRRK2 in the proliferation of neural precursors and in adult neurogenesis are promising but at an early stage (Milosevic et al., 2009; Winner et al., 2011).

In conclusion, there is a remarkable degree of overlap between the effects of Wnt signaling and LRRK2 on neurogenesis. The importance of LRRK2 in canonical Wnt signaling further supports the notion of a specific function for LRRK2 in Wnt-mediated neurogenesis. This hypothesis can be investigated by crossing the relevant transgenic animals with known defects in Wnt-mediated neuronal differentiation with LRRK2 transgenics looking for an enhancement or rescue of phenotype. Of course, such experiments come with the usual important caveats associated with using animal models. For example, long non-coding RNAs are known to be poorly conserved between species making their study in model organisms of questionable relevance to humans (Pang et al., 2006). Human-specific transcriptional networks have also been reported in the brain (Konopka et al., 2012). However, at the level of protein function, conservation across species is usually very high, and thus, even though the data should be treated with caution, crossing of transgenic animal models is a justifiable approach. Indeed, this strategy has proven particularly useful for unveiling milder neurodevelopmental phenotypes. For example, crossing of *Wnt7a* and *Dvl1* knockout mice allowed a requirement for these genes in the development of cerebellar glomerular rosettes to be uncovered (Ahmad-Annuar et al., 2006). As *Lrrk2* knockout and familial *PARK8* mutant transgenic mice likely represent models of subtly increased and decreased canonical Wnt signaling, respectively, crossing of these lines with *Wnt7a* and/or *Dvl1* knockout animals would be of great interest.

## Conflict of Interest Statement

The authors declare that the research was conducted in the absence of any commercial or financial relationships that could be construed as a potential conflict of interest.
